# Hypertriglyceridemic Pancreatitis: Is Insulin Monotherapy A Feasible Therapeutic Option?

**DOI:** 10.7759/cureus.3461

**Published:** 2018-10-17

**Authors:** Faisal Inayat, Fahad Zafar, Iqra Riaz, Fariha Younus, Asad S Baig, Zahid Imran

**Affiliations:** 1 Internal Medicine, Allama Iqbal Medical College, Lahore, PAK; 2 Internal Medicine, King Edward Medical University, Lahore, PAK; 3 Cardiology, Einstein Healthcare Network, Philadelphia, USA; 4 Internal Medicine, Services Institute of Medical Sciences, Lahore, PAK; 5 Internal Medicine, Nawaz Sharif Medical College, Gujrat, PAK; 6 Internal Medicine, Doctor's Hospital, Gujrat, PAK

**Keywords:** hypertriglyceridemia, acute pancreatitis, insulin monotherapy, diagnosis, management

## Abstract

Hypertriglyceridemic pancreatitis (HTGP) is an uncommon but well-established clinical entity. Although the initial clinical features are similar to pancreatitis due to other etiologies, the severity of the disease and the risk of complications are higher in these patients. Prompt diagnosis and appropriate treatment are crucial in patients with hypertriglyceridemia-induced pancreatitis to avoid life-threatening complications. The initial conservative treatment is applied followed by additional specific therapies tailored to decrease serum triglyceride levels. This includes plasmapheresis, insulin, heparin infusion, and hemofiltration. After the acute episode, lifestyle modifications along with hypolipidemic medications should be initiated to prevent further events. Currently, there is paucity of the medical literature directly comparing different treatment modalities. This article illustrates the use of insulin therapy for HTGP as a feasible therapeutic choice. Randomized controlled trials are warranted to outline a generalized and efficient treatment for this serious disorder.

## Introduction

Acute pancreatitis is a potentially life-threatening inflammation of the pancreas with a wide constellation of etiologies. Alcoholism and gallstones are the most common causes while abdominal surgery, certain medications, cystic fibrosis, hypercalcemia, hyperparathyroidism, infection, injury to the abdomen, and malignancy are among the less common etiologies [1-3]. Hypertriglyceridemic pancreatitis (HTGP) is relatively uncommon with an estimated incidence up to 10% of all the cases [4]. Although the initial clinical presentation corresponds to that of acute pancreatitis following other etiologies, HTGP has been associated with higher severity and increased complication rate. In the current times, no specific treatment guidelines are available. However, insulin, heparin, fibric acids, and omega-three fatty acids have been used to treat HTGP either as monotherapy or in variable combinations [5]. Plasmapheresis has also been employed in patients with HTGP but it has been associated with myriad complications. Furthermore, the widespread availability of this modality may pose a problem [6]. After treating the acute phase of HTGP, lifestyle modifications such as dietary fat and sugar restriction, regular exercise, weight loss, blood sugar control, and lipid-lowering drugs are imperative for the long-term management of HTGP as well as prevention of recurrence of the disease.

The present study describes a patient having severe HTGP treated effectively with insulin monotherapy. No complications were encountered and the patient demonstrated an excellent recovery. Therefore, insulin monotherapy may be a feasible treatment choice in patients with HTGP, especially in clinical settings that lack plasmapheresis. Additionally, we review the pertinent medical literature for the epidemiology, clinical features, comorbid conditions, and diagnostic investigations, along with a special emphasis on various management options available for this condition.

## Case presentation

A 39-year-old male presented to our medical center with acute-onset sharp abdominal pain for four days. The pain was continuous, radiating to the back, and it was associated with nausea. The patient had a past medical history significant for gout, pre-diabetes, and hyperlipidemia. He was not on any medications and was educated to control the metabolic abnormalities only with diet and exercise. He was married and worked as a chef. He denied tobacco, alcohol, or illicit drug use. His family history was negative for metabolic syndromes and lipid abnormalities. On presentation, physical examination was remarkable for epigastric tenderness. He appeared dehydrated and diaphoretic, febrile to 101.2° F, and tachycardic to 114 beats per minute.

Laboratory parameters were remarkable for hypertriglyceridemia, hyperglycemia, and markedly elevated serum lipase levels. The details of his laboratory evaluations are provided (Table 1).

**Table 1:** Initial laboratory investigations of the patient with respective reference ranges.

Laboratory parameter	Specimen	Patient result	Reference range
White cell count	Serum	13.92	4.5-11.0/uL
Hemoglobin	Serum	12.1	13-18 g/dL
Hematocrit	Serum	34.9	40%-52%
Platelets	Serum	181×10^3^	150-450× 10^3^/uL
Triglyceride	Serum	5047	<150 mg/dL
Cholesterol	Serum	499	<200 mg/dL
HbA1c	Serum	11.8	4%-5.6%
Random blood glucose	Serum	467	72-99 mg/dL
C-reactive protein	Serum	270	<1.0 mg/L
Serum lipase	Serum	4397	0-50 U/L
Blood urea nitrogen	Serum	9	7-20 mg/dL
Creatinine	Serum	0.7	0.4-1.2 mg/dL

**Figure 1:**
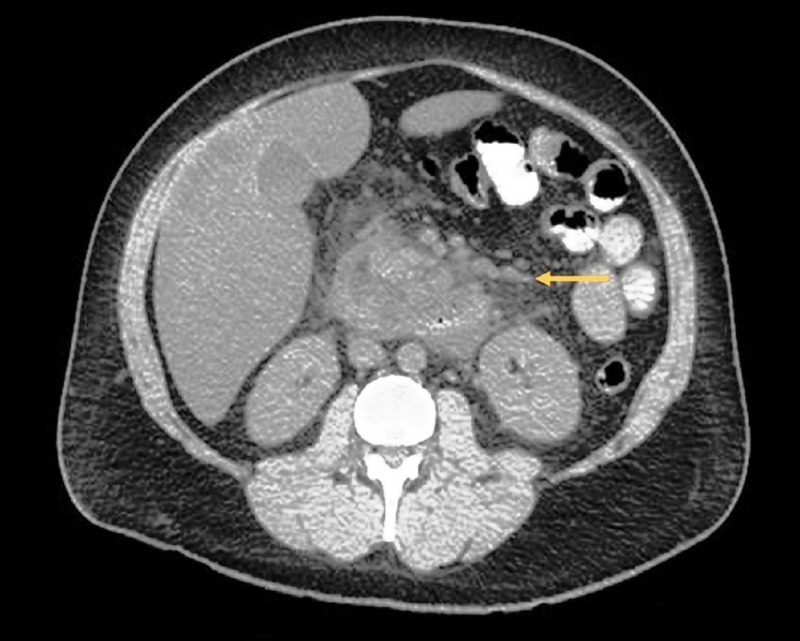
Computed tomography abdomen showing enlarged and edematous pancreas with smooth, indistinct margins, nonvisualized pancreatic duct, and peripancreatic fat stranding. Arrow indicates the above-mentioned findings.

Serum electrolytes, liver and renal function tests, coagulation profile, and lactate dehydrogenase were within normal limits. Computed tomography abdomen demonstrated peripancreatic fatty infiltration and moderate edema, suggestive of acute pancreatitis (Figure 1).

There was no evidence of gallstones. Therein, the probable causes for patient’s disease were systematically excluded, and he was eventually diagnosed with HTGP based on the detailed clinical history, physical examination, laboratory parameters, and radiological findings.

The patient was admitted to the medical intensive care unit. Conservative treatment was initiated with intravenous hydration utilizing Ringer’s lactate and morphine for pain control. As therapeutic apheresis for hypertriglyceridemia was not available, he was initiated on insulin infusion 0.1 units/kg/hour along with 75 cc/hour intravenous sugar solution (dextrose 5% in water). After one day of insulin treatment, his triglyceride level trended down to 3894 mg/dL (normal, <150 mg/dL). He was continued on insulin infusion and dextrose water with hourly blood glucose monitoring. After 12 days of intensive insulin monotherapy, the triglyceride level decreased to less than 500 mg/dL. The patient recovered well with insulin treatment with diminution of symptoms. After he resumed enteral feeds, he was discharged from the hospital in stable condition on a long-term fenofibrate therapy (TriCor®; AbbVie Inc., North Chicago, IL). Furthermore, he was also referred for genetic testing to rule out genetic disorders associated with his metabolic lipid aberration. At the one-month follow-up, the patient showed complete recovery of the symptoms with normal biochemical profile.

## Discussion

Hypertriglyceridemia is a well known but underestimated cause of acute pancreatitis. It is considered a relatively uncommon etiology of acute pancreatitis after gallstones and alcohol abuse [1]. Hypercalcemia, iatrogenic injuries in procedures like endoscopic retrograde cholangiopancreatography (ERCP), genetic mutations, drugs, infections, toxins, trauma, abdominal surgery, malignancy, and idiopathic are included among the less common causes [2-3]. According to one estimate, hypertriglyceridemia accounts for up to 10% of all the pancreatitis episodes while in pregnant patients, it may lead to pancreatitis in more than 50% of all cases [4]. The underlying causes of hypertriglyceridemia can be either primary or secondary. Primary HTGP is due to genetic mutations leading to dyslipidemias. Several secondary factors have the propensity to cause HTGP, including poorly controlled diabetes mellitus, alcohol abuse, pregnancy, and medications such as oral estrogens, tamoxifen, propofol, valproate, isotretinoin, clomiphene, beta blockers, protease inhibitors, and mirtazapine [5]. It is essential to determine the underlying etiology in order to decide about the appropriate management.

The exact pathogenesis of HTGP is controversial to date. The most commonly accepted hypothesis implicates the excess of triglycerides that are subsequently hydrolyzed by pancreatic lipases to release free fatty acids (FFAs). These FFAs exceed the binding capacity of albumins that have detergent properties. They attack pancreatic acinar cell, platelets, and vascular endothelial cells. This results in ischemia leading to the acidic environment, which further worsens the FFA toxicity [6-7]. Furthermore, the burden of triglycerides increases the blood viscosity and impairs the vascular flow to the pancreas, resulting in further ischemia and pancreatic injury. Hypertriglyceridemia also contributes to acute pancreatitis by aggravating the endoplasmic reticulum stress [7].

The clinical presentation of HTGP is similar to acute pancreatitis secondary to other causes. The only difference is the age group as the patients with HTGP tend to be relatively younger [8]. The initial clinical manifestation is mostly related to the abdominal pain, which is present in approximately 95% of cases [9]. It can be a generalized pain in the upper abdomen or localized to the epigastrium, right hypochondrium or rarely to left hypochondrium. It often radiates to the back and may be exacerbated by eating and drinking. It is notable that leaning forward may relieve the pain by reducing the stretch on the pancreas. Apart from the abdominal pain, other important symptoms include nausea and vomiting. The clinical examination findings include upper abdominal tenderness, guarding, epigastric distension, hypoactive bowel sounds, tachycardia, hypotension, low-grade fever, and shallow rapid respiration. The less common findings include rebound tenderness, pleural effusions, subcutaneous fat necrosis, Cullen sign, and Gray Turner’s sign. These uncommon findings usually reflect the complications of acute pancreatitis [9].

The diagnosis of acute pancreatitis is based on the presence of at least two of the following features: (a) typical abdominal pain, (b) elevated pancreatic enzymes two-to-three times above the normal values, and (c) radiological findings showing acutely inflamed pancreas. The diagnosis of hypertriglyceridemia as a cause of acute pancreatitis is challenging as mild elevations in serum triglyceride levels are observed in all cases of acute pancreatitis, irrespective of the underlying etiology [10]. A markedly elevated triglyceride level i.e. >1000 mg/dL in a case of acute pancreatitis provides strong evidence of HTGP. The triglyceride level of 500 mg/dL or more should raise a high-degree suspicion for HTGP, especially in the absence of other probable etiologies. It is essential to assess the serum triglyceride levels within 24 hours of presentation as the delayed evaluations may show falsely low levels either due to fasting or owing to intravenous fluid administration. Moreover, it is notable that high triglyceride levels may also result in falsely low levels of pancreatic enzymes due to the interference in the enzyme assays. After diagnosing HTGP, it is important to establish the cause of hypertriglyceridemia such as thyroid function tests to exclude endocrine causes and other specific tests to exclude familial causes. Similarly, measurement of complete blood count, C-reactive protein, serum glucose, serum calcium, serum electrolytes, renal function testing, lactate dehydrogenase, blood urea nitrogen, and creatinine is important to determine the severity and prognosis of HTGP [11].

Currently, no specific guidelines are available for the treatment of HTGP. The basic approach follows the immediate stabilization of the patients and then, the long-term management is initiated. The acute phase of HTGP must be treated urgently as it can lead to life-threatening conditions such as the multiple organ dysfunction syndrome (MODS) and/or the systemic inflammatory response syndrome (SIRS). Therefore, aggressive initial management is of paramount importance to reduce the morbidity and mortality in these patients. The initial treatment of HTGP includes restriction of oral intake, administration of intravenous fluids, analgesics, as well as antibiotics in selected patients. Following the initial resuscitation, the therapeutic approaches aim to reduce the serum triglyceride levels. Heparin infusion, insulin therapy, a combination of heparin and insulin infusion, and/or plasmapheresis have previously been used in this regard. Once the acute phase of HTGP is over, the recurrence is avoided by either pharmacological interventions such as oral anti-hyperlipidemic agents (fenofibrates, nicotinic acid, or omega-three fatty acids) or nonpharmacological interventions like weight loss, dietary-fat restriction, and strict glycemic control in diabetic patients [11]. The severity of acute pancreatitis is assessed based on the APACHE-II scale and Balthazar grade. The patients with APACHE-II score of more than eight or Balthazar grade E are managed in the intensive care unit [12]. Nasogastric feeding and parenteral nutrition with a fat-free diet are recommended in such patients due to a prolonged clinical course. 

Prompt lowering of serum triglyceride levels is essential for the management of HTGP. It is either achieved by plasmapheresis alone or in a combination with intensive insulin/heparin therapy [13]. As plasmapheresis is not readily available and HTGP frequently presents in diabetic patients, insulin is beneficial as it lowers both the serum triglyceride and blood glucose levels. Heparin releases lipoprotein lipase from the endothelial cells into circulation. This enzyme accelerates the degradation of chylomicrons, which is followed by a decline in the serum triglyceride levels. However, prolonged heparin infusions deplete the lipoprotein lipase that eventually may culminate in hypertriglyceridemia. Additionally, heparin infusion may also increase the risk of pancreatic necrosis and hemorrhage [14-15]. Therefore, the use of heparin in HTGP is controversial. Insulin monotherapy, on the other hand, activates endothelial lipoprotein lipase, which clears the plasma triglyceride and hence, lowers the serum triglyceride levels. Insulin can be administered subcutaneously or intravenously, although intravenous insulin is preferred due to its easy titration. Continuous insulin infusion as monotherapy is effective for HTGP in both categories of patients with diabetics and nondiabetics [16]. Apart from the monotherapy, insulin can also be used in combination with plasmapheresis. This combination therapy has a higher efficacy in reducing serum triglyceride levels but at the cost of higher rate of complications such as respiratory failure and acute kidney disease [11, 17]. The treatment of HTGP in pregnant patients is almost the same as in nonpregnant patients. Oral anti-hyperlipidemic drugs are avoided in pregnancy while omega-three fatty acids and plasmapheresis are considered to be the first-line treatment options [18-19].

## Conclusions

Although insulin, heparin, and plasmapheresis have been used, no specific therapeutic guidelines exist for severe HTGP. The present study highlights insulin monotherapy as an effective treatment option with promising outcomes, particularly in the clinical settings with no plasmapheresis. Furthermore, it prompts concerned clinicians to undertake comprehensive studies to establish an optimal treatment for patients with HTGP.
